# Improved Analytical Sensitivity of Lateral Flow Assay using Sponge for HBV Nucleic Acid Detection

**DOI:** 10.1038/s41598-017-01558-x

**Published:** 2017-05-02

**Authors:** Ruihua Tang, Hui Yang, Yan Gong, Zhi Liu, XiuJun Li, Ting Wen, ZhiGuo Qu, Sufeng Zhang, Qibing Mei, Feng Xu

**Affiliations:** 1 School of Life Sciences, Northwestern Polytechnical University, Xi’an, 710072 P.R. China; 2 Key Laboratory for Space Bioscience and Biotechnology, Northwestern Polytechnical University, Xi’an, 710072 P.R. China; 3 Bioinspired Engineering and Biomechanics Center (BEBC), Xi’an Jiaotong University, Xi’an, 710049 P.R. China; 4 The Key Laboratory of Biomedical Information Engineering of Ministry of Education, School of Life Science and Technology, Xi’an Jiaotong University, Xi’an, 710049 P.R. China; 5 Xi’an Diandi Biotech Company, Xi’an, 710049 P.R. China; 6 Key Laboratory of Thermo-Fluid Science and Engineering of Ministry of Education, School of Energy and Power Engineering, Xi’an Jiaotong University, Xi’an, 710049 P.R. China; 7 Department of Chemistry, University of Texas at El Paso, 500 West University Ave, El Paso, Texas 79968 USA; 8 College of Bioresources Chemical and Materials Engineering, Shaanxi University of Science and Technology, Xi’an, 710021 China

## Abstract

Hepatitis B virus (HBV) infection is a serious public health problem, which can be transmitted through various routes (*e*.*g*., blood donation) and cause hepatitis, liver cirrhosis and liver cancer. Hence, it is necessary to do diagnostic screening for high-risk HBV patients in these transmission routes. Nowadays, protein-based technologies have been used for HBV testing, which however involve the issues of large sample volume, antibody instability and poor specificity. Nucleic acid hybridization-based lateral flow assay (LFA) holds great potential to address these limitations due to its low-cost, rapid, and simple features, but the poor analytical sensitivity of LFA restricts its application. In this study, we developed a low-cost, simple and easy-to-use method to improve analytical sensitivity by integrating sponge shunt into LFA to decrease the fluid flow rate. The thickness, length and hydrophobicity of the sponge shunt were sequentially optimized, and achieved 10-fold signal enhancement in nucleic acid testing of HBV as compared to the unmodified LFA. The enhancement was further confirmed by using HBV clinical samples, where we achieved the detection limit of 10^3^ copies/ml as compared to 10^4^ copies/ml in unmodified LFA. The improved LFA holds great potential for diseases diagnostics, food safety control and environment monitoring at point-of-care.

## Introduction

Hepatitis B virus (HBV) infection is a serious public health problem in the world^1^, which can cause hepatitis, liver cirrhosis and liver cancer. Unfortunately, 240 million people are influenced by HBV infection^2^ with nearly 1 million deaths every year^3^. HBV infection can be transmitted through different routes such as mother to child, donation blood, sexual contact, intravenous drug use and medical exposure^4^. Thus, it is essential to do rapid screening of high-risk HBV patients in blood donation for preparing better infection control strategies and therapeutic suggestions. At present, several technologies have been used for HBV detection, including immunoassays (*e*.*g*., enzyme-linked immunosorbent assay (ELISA)^5^ and lateral flow immunoassay (LFIA)^6^) and nucleic acid testing (NAT) technologies (*e*.*g*., polymerase chain reaction (PCR), real-time quantitative PCR (qPCR)^7^). However, there are several challenges associated with protein-based immunoassays, such as large sample volume, antibody instability and poor specificity^8^. Although NAT technologies have advantages of good specificity and stability, they are high cost, complex, time consuming due to the need of large equipment and skilled workers, restricting their widespread applications, especially in resource-poor settings. More recently, nucleic acid hybridization-based lateral flow assay (LFA) as a simple, rapid, low-cost, stable easy-to-use tool has attracted significantly increasing attention for disease diagnosis^9, 10^. However, the poor analytical sensitivity of LFA restricts its application for HBV diagnosis^11^, especially considering the low concentration range of HBV nucleic acid in clinical samples (10^3^–10^9^ copies/ml)^12^. Hence, there is an urgent to develop a highly sensitive LFA for HBV detection.

Nowadays, several methods have been used to improve the analytical sensitivity of lateral flow assay. For example, chemical methods such as probe-based method^13^, enzyme-based method^9^, paper-based dialysis concentration^14^ and physical methods such as wax-based method^15^, evaporative concentration^16^. There are instability, complex preparation progress or operations and external power supply due to need high-cost chemical reagents and portable device. Considering the LFA analytical sensitivity is highly dependent on the reaction time between target and AuNP-DPs^17–20^, several methods have been used to control the flow rate of liquid to improve the LFA analytical sensitivity through altering the shape of LFA sample pad, the wetting distance of liquid and pore size of paper. For instance, irregular size architecture of sample pad and a larger strip have been used to improve analytical sensitivity and achieved 10-fold^21^. However, such changes significantly increase the needed sample volume. Additionally, PDMS-paper^18^ and hydrogel-paper hybrid material^19^ have been integrated into conventional LFAs as a shunt for improving LFA analytical sensitivity through increasing the reaction time of the fluid because of the inverse relationship between the LFA analytical sensitivity and the reaction time^8–10, 12^. However, such hybrid material shunts need complex preparation process and more reagents. Therefore, a low-cost, simple and easy-to-use method for enhancing analytical sensitivity of LFA is urgent to develop for improving analytical sensitivity. Universally, sponge as a paper-like material has good wicking action and good hydrophilic property, which has been utilized for designing multiple types of valves to control the fluid flow in paper-based microfluidic devices avoiding complex operations^22^. Therefore, sponge as a promising porous material could be incorporated into LFA to control flow rate for improving its analytical sensitivity.

**Figure 1:**
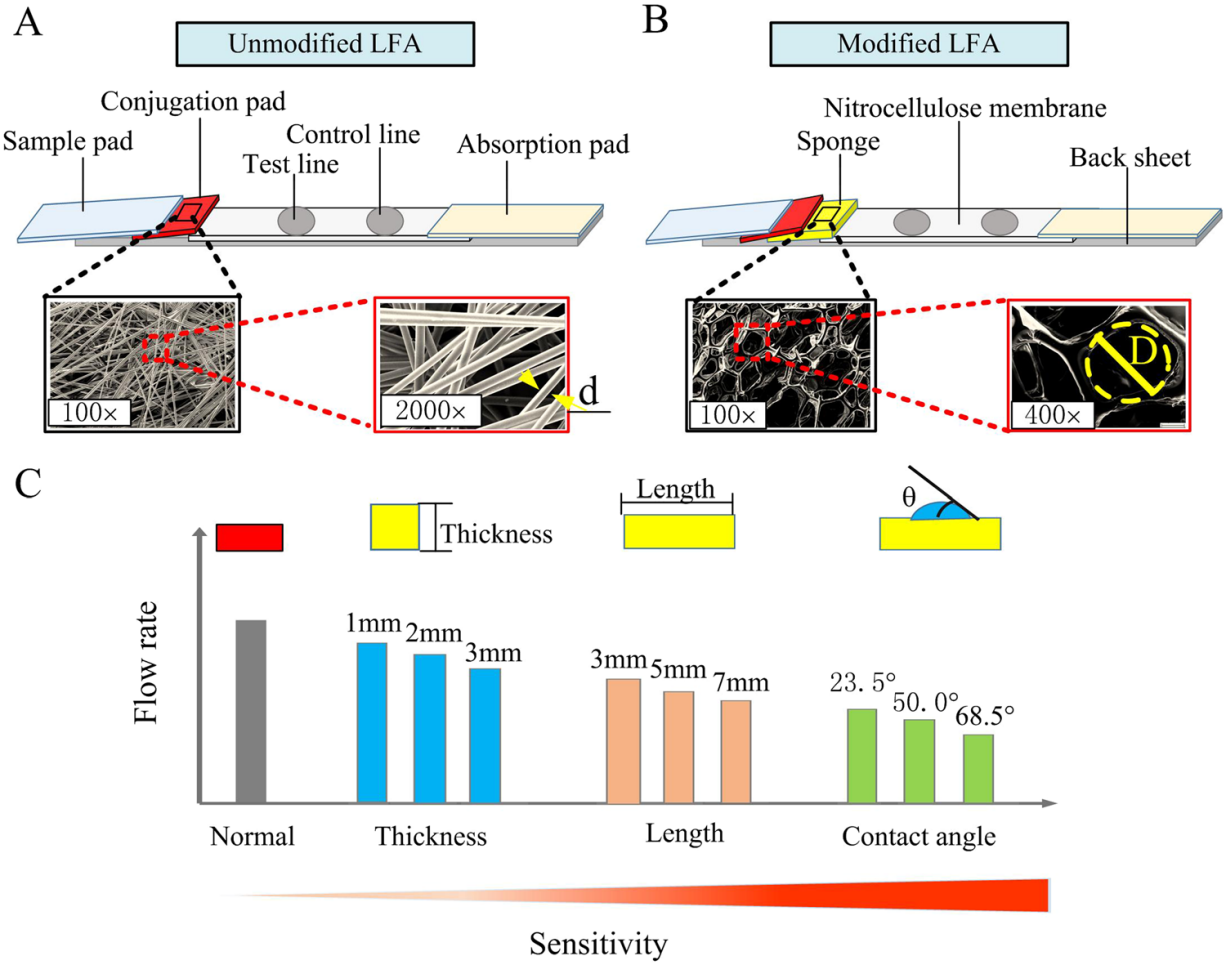
The schematic of improvement the analytical sensitivity of LFA by sponge. The schematic of the unmodified LFA (**A**), the modified LFA (**B**) and the optimization parameters of sponge shunt (**C**).

In this study, sponge shunt was integrated into LFA to decrease the fluid flow rate and thus to enhance its analytical sensitivity (Fig. 1). We successfully achieved 10-fold signal enhancement in nucleic acid testing of HBV by optimizing the thickness, length and hydrophobicity of sponge. With this, the detection limit as low as 10^3^ copies/ml for HBV clinical samples has been obtained as compared to the detection limit of 10^4^ copies/ml in unmodified LFAs. We envision that our enhanced LFAs hold great potential for detections of other targets in point of care testing (POCT).

## Experimental methods

### Materials

HAuCl_4_.4H_2_O was purchased from Sinnopharm Chemical Reagent Co., Ltd. (Shanghai, China). Tween 20, Tris (2-carboxyethyl)-phosphine (TCEP) and streptavidin were purchased from Sigma-Aldrich (St. Louis, Mo, USA). Trisodium phosphate, ethylene diamine tetraacetic acid (EDTA), sodium chloride were bought from Tianli Chemical reagent Co., Ltd. (Tianjin, China). 20 × SSC buffer was bought from Ambion Co., Ltd. (USA). HBV probes were synthesized from Sangon Biotechnology Co., Ltd. (Shanghai, China). Sponge was bought from local store (Yunli Sponge Producers Company, Shenzhen, China). The brand was hydroponic sponge, which is composed of polyurethane material, the porosity is 50%, the size is 20 mm × 10 mm × 100 mm (length × width × height). HBV positive serum was purchase from Daan gene (Guangzhou, China) Co., LTD. FTA card was bought from Whatman (USA). Nitrocellulose membrane (Millipore HFB18002, USA), backing pad, absorbent pad, conjugate pad and sample pad were supplied by Jiening Biotech Co., Ltd. (Shanghai, China). IsoAmp®Ш Universal tHDA Kit was bought from New England Biolabs (NEB, USA). All chemicals used in this study were analytical reagent grade.

### Preparation of lateral flow test strip

According to the previous studies^14^, AuNP particles of 13 nm diameter were prepared. Similarly, the AuNP-detector probe conjugates, test line and control line with a slight modification were also prepared based on the reported studies^14^. Then, HBV probes were used to modify AuNP according to the preS1 sequence of HBV complete genome (Genbank: X98077.1). Please see the detailed sequences in Supplementary Information Table S1. Then, absorbent pad (length × width × height: 25 mm × 3 mm × 0.668 mm), nitrocellulose (NC) membrane (length × width × height: 20 mm × 3 mm × 0.01 mm), sponge (length × width × height: 7 mm × 3 mm × 3 mm), conjugate pad (length × width × height: 10 mm × 3 mm × 0.2 mm) and sample pad (length × width × height: 15 mm × 3 mm × 0.8 mm) were sequentially mounted on a plastic adhesive backing pad with 2 mm overlap between each two adjacent pads according to Fig. 1.

### Optimization assay

In a validation experiment, different synthesis target concentrations of HBV nucleic acid (50 nM, 25 nM, 10 nM, 5 nM, 2.5 nM, 1 nM, 0.5 nM, 0.25 nM, 0.1 nM, 0 nM) were used for optimization assay. Firstly, different thicknesses of sponge (1 mm, 2 mm, 3 mm, 4 mm) with 5 mm length and 50° contact angle of sponge shunt were optimized. On this basis of optimal thickness, different lengths of sponge (3 mm, 5 mm, 7 mm, 9 mm) with 3 mm thickness and 50° contact angle sponge shunt (20% tween 20 was used to treat sponge) were also optimized. Additionally, different concentrations of TritonX-100 (30%, 20%, 10%, 5%) were used to treat sponge to change its contact angle with 3 mm thickness and 7 mm length sponge shunt, the contact angle were measured by Keyence VHX-100 microscope and Image J software. To validate our assay, we performed three replicates for the optimization experiments for high concentrations (2.5–50 nM) and eight replicates for low concentrations (0.05–1 nM), according to the ref. 23.

### Mathematic simulation

To reveal the underlying mechanism of the delaying effect of the sponge with varying geometric and hydrophilic properties, a mathematic model is developed based on the electrical circuit analogues method, which describes the liquid wicking behavior in a sequential contacted strips on the base of Darcy’ law^24, 25^ (Fig. S1). Generally, the capillary force *P*_ca_ treats as the main driven force is analogous to the voltage *V* in the electrical circuit. The wicking speed *Q* and fluidic resistance $\frac{\mu {L}_{{\rm{i}}}}{{K}_{{\rm{i}}}{W}_{{\rm{i}}}{H}_{{\rm{i}}}}$ are regarded as the electrical current *I* and resistance *R*_*i*_, respectively, where *μ* is the viscosity and the material parameters, such as the length *L*, width *W*, height *H*, permeability *K* of strips, are experimentally measured and theoretically calculated by our previous method^17, 18^ (Table S2). Considering that liquid is quickly added into the sample pad by manual pipetting in experiment, the sample pad is fully wetted and regarded as a liquid pool to supply the sample for connected conjugated pad. Additionally, the liquid passes through the absorption pad has no significant effect on the LFA analytical sensitivity, so the end of NC membrane is regarded as the cut-off line. For simplification, it is assumed that the main liquid transport process in simulation begins from conjugated pad (1) to sponge pad (2), then to NC membrane (3). Then, the liquid transport process in the assembly is represented as an electrical circuit (Fig. S1). Compared with the Ohm’s law (*I* = *V*/*R*), the wicking speeds in different pads are evaluated as following,

1 $${Q}_{1}={\varepsilon }_{1}{W}_{1}{H}_{1}\frac{\partial {L}_{1}(t)}{\partial t}=\frac{{P}_{{\rm{ca1}}}}{\mu /({K}_{1}{W}_{1}{H}_{1})\cdot {L}_{1}(t)}.$$

2 $${Q}_{2}={\varepsilon }_{2}{W}_{2}{H}_{2}\frac{\partial {L}_{2}(t)}{\partial t}=\frac{{P}_{{\rm{ca2}}}}{\frac{\mu {L}_{1}}{{K}_{1}{W}_{1}{H}_{1}}+\frac{\mu {L}_{2}(t)}{{K}_{2}{W}_{2}{H}_{2}}}.$$

3 $${Q}_{3}={\varepsilon }_{3}{W}_{3}{H}_{3}\frac{\partial {L}_{3}(t)}{\partial t}=\frac{{P}_{{\rm{ca3}}}}{\frac{\mu {L}_{1}}{{K}_{1}{W}_{1}{H}_{1}}+\frac{\mu {L}_{2}}{{K}_{2}{W}_{2}{H}_{2}}+\frac{\mu {L}_{3}(t)}{{K}_{3}{W}_{3}{H}_{3}}}.$$

The above equations can be solved by integration as following,

4 $${L}_{1}(t)=\sqrt{\frac{2{K}_{1}{P}_{{\rm{ca1}}}}{\mu {\varepsilon }_{1}}\cdot t}.$$

5 $${L}_{2}(t)=\sqrt{\frac{2{K}_{2}{P}_{{\rm{ca2}}}}{\mu {\varepsilon }_{2}}\cdot (t-{t}_{1})+{(\frac{{K}_{2}{W}_{2}{H}_{2}}{{K}_{1}{W}_{1}{H}_{1}}\cdot {L}_{1})}^{2}}-\frac{{K}_{2}{W}_{2}{H}_{2}}{{K}_{1}{W}_{1}{H}_{1}}\cdot {L}_{1}.$$

6 $${L}_{3}(t)=\sqrt{\frac{2{K}_{3}{P}_{{\rm{ca3}}}}{\mu {\varepsilon }_{3}}\cdot (t-{t}_{2})+{(\frac{{K}_{3}{W}_{3}{H}_{3}\cdot {L}_{1}}{{K}_{1}{W}_{1}{H}_{1}}+\frac{{K}_{3}{W}_{3}{H}_{3}\cdot {L}_{2}}{{K}_{2}{W}_{2}{H}_{2}})}^{2}}-(\frac{{K}_{3}{W}_{3}{H}_{3}\cdot {L}_{1}}{{K}_{1}{W}_{1}{H}_{1}}+\frac{{K}_{3}{W}_{3}{H}_{3}\cdot {L}_{2}}{{K}_{2}{W}_{2}{H}_{2}}).$$

To solve these equations, it’s important to obtain the capillary force, which is hardly to be determined experimentally. Generally, paper strip is assumed to be composed by extensive parallel capillaries^26^, and the strip capillary force has a relationship with the sum of the capillary forces in every capillary tube, giving as following,

7 $${P}_{ca}\propto \pi d\sigma \,\cos \,\theta (\frac{W}{d}{\varepsilon }^{1/3})(\frac{H}{d}{\varepsilon }^{1/3})/(\varepsilon WH).$$

Where the strip pore radius *d* and the porosity *ε* are also obtained by experimental method. The liquid is regarded as water, and its parameters such as surface tension *σ*, contact angle *θ* are shown in Table S3. The capillary forces are determined by monitoring the liquid velocity through the above three materials, respectively. Thus, the liquid wicking distance *L* can be simply obtained from the above equations.

### Clinical sample detection

Clinical samples were obtained by the First Affiliated Hospital of Xi’an Jiaotong University. Informed consent for research use of blood was sought and obtained from each study participant. The study protocol was approved by the Institute Research Ethics Committee of the First Affiliated Hospital of Xi’an Jiaotong University. The methods were performed in accordance with the approved guidelines. Blood samples were collected from 12 patients with clinically confirmed HBV infection and quantified using qPCR according to the published protocol^27^. (Please see the detailed information in Supplementary Information). The concentrations of HBV clinical samples were provided by the hospital (The detail was in Table S4). In this study, the initial concentration of positive HBV serum was 10^7^ copies/ml which was diluted with negative serum to the concentration of 10^6^ copies/ml, 10^5^ copies/ml, 10^4^ copies/ml, 10^3^ copies/ml, 10^2^ copies/ml, 10^1^ copies/ml, 10^0^ copies/ml to detect the analytical sensitivity of LFA. Then, 30 μL HBV blood or serum was utilized to extract DNA using FTA card. The single stranded nucleic acid sequence of HBV was amplified by asymmetric Helicase-Dependent Amplification (tHDA) method. According to the instruction of tHDA primer design^28^, the primers of the preS1 gene sequence of HBV were designed (The detailed information was in Supplementary Information Table S1). The amplification reaction volume was 50 μL depended on the instructions with the slight modifications^28^, including 5 μL of 10 × annealing buffer, 2 μL of 100 mM MgSO_4_, 4 μL of 500 mM NaCl, 3.5 μL of IsoAmp® dNTP solution, 5 μL of 100 μM Forward primer, 1 μL of 5 μM Reverse primer, 6 μL of IsoAmp® Ш Enzyme Mix, 23.5 μL of ddH_2_O and the template of FTA card. After tHDA amplification, 50 μL running buffer of LFA was added into the amplification product and then used for LFA detection. After 15 min, the result was observed by the naked eye. The images were captured by iPhone 6S, and the optical densities of test strips were measured by Image-Pro Plus 6.0 software. To assure that the modified assay has a decreased detection limit for clinical samples as compared to the unmodified LFA, eight replicates were performed for each test of positive samples with HBV concentrations in the range of 10^5^–10^2^ copies/ml.

## Results and Discussion

To develop a low-cost, simple and easy-to-use LFA for HBV detection with enhanced analytical sensitivity, a new LFA structure was designed by integrating sponge shunt into conventional LFA (Fig. 1). Compared with the structure of unmodified LFA (Fig. 1A **)**, the sponge shunt was added in the modified LFA between the conjugation pad and nitrocellulose membrane, which can delay the fluid flow rate to improve the analytical sensitivity^18–20^, because the radius of sponge structure was larger than that of conjugation pad, making the capillary force of sponge less than that of conjugation pad^29^ (Fig. 1B).

**Figure 2:**
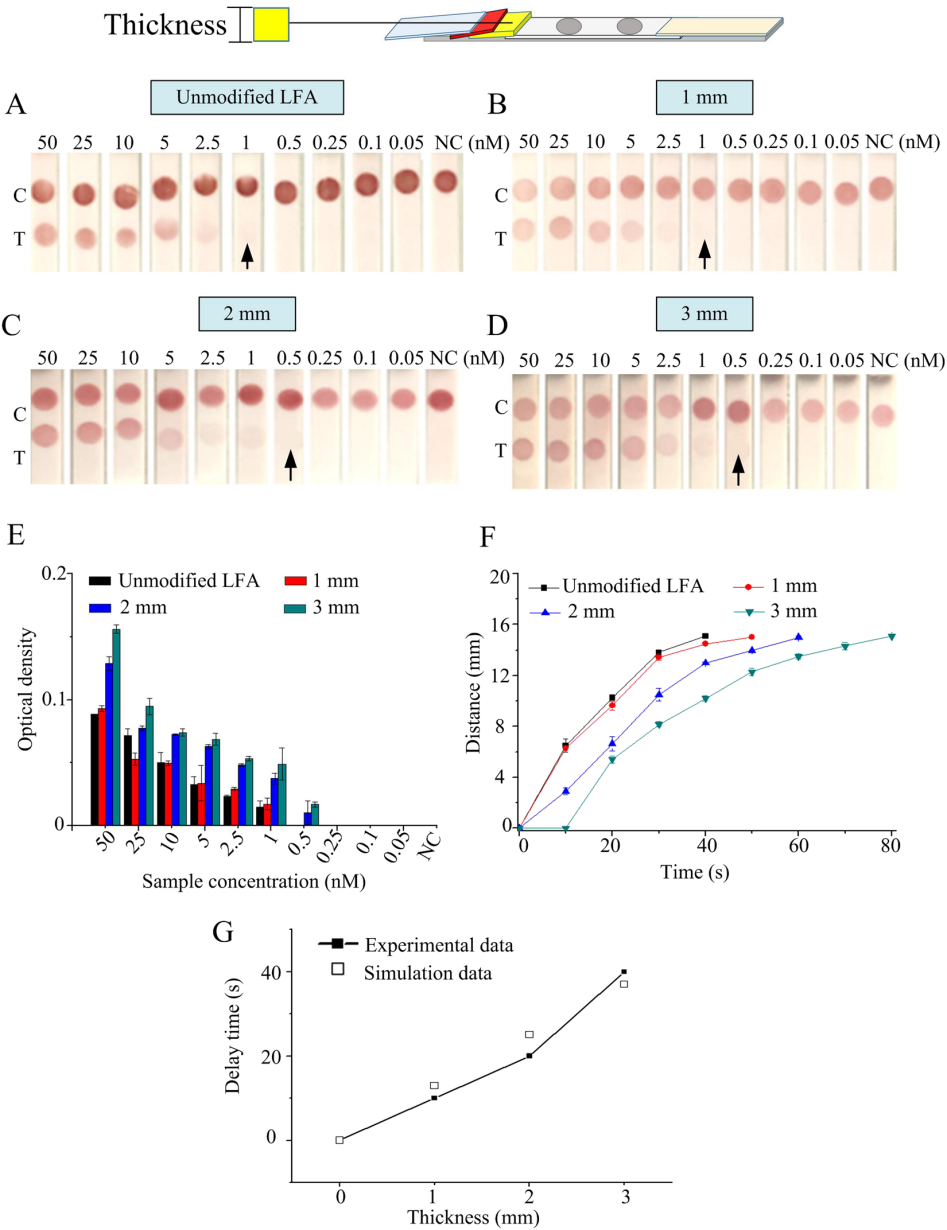
Analytical sensitivity improvement of LFA by different thickness of the sponge. The detection limit of unmodified LFA is 1 nM (**A**), 1 mm of sponge thickness is 1 nM (**B**), 2 mm of sponge thickness is 0.5 nM (**C**) and 3 mm of sponge thickness is 0.5 nM (**D**). (**E**) The optical density of LFA test line under different thicknesses of sponge. (**F**) The wicking distance on the NC membrane with time at different sponge shunt thicknesses. (**G**) The relationship between the experimental data and simulation data. Three replicates were performed for the optimization experiments for high concentrations in the range of 2.5–50 nM (*N* = 3), while eight replicates were performed for lower concentrations in the range of 0.05–1 nM (*N* = 8).

To achieve the analytical sensitivity enhancement, different parameters of the sponge shunt were optimized according to the sequence of Fig. 1C. Firstly, different thicknesses of sponge (*i*.*e*., 1–4 mm) with 5 mm length and 50°contact angle of sponge shunt were changed, Fig. 2. The results indicate that the detection limit of unmodified LFA is 1 nM (Fig. 2A), which is improved to 0.5 nM by using sponge with thickness of 2 mm and 3 mm though no improvement is observed in the 1 mm thick sponge case (Fig. 2B–D). The optical density of the detection line in the LFA was further quantified, the result shows that the optical density of the 3 mm thick sponge is higher than that of 2 mm thick sponge (Fig. 2E). The thicker sponge (4 mm) was also tried, but the result indicates that the liquid cannot flow through NC membrane within 15 minutes, because the flow rate of fluid is too slow. Additionally, The wicking distance on the NC membrane with time was measured, the result shows that the wicking distance increases gradually with increasing sponge thickness. Compared with the unmodified LFA, the time for wicking the whole NC membrane of the LFA integrated with 3 mm thick sponge shunt is delayed for 40 seconds. Such a flow delay can increase the reaction time between targets in sample and AuNP-DPs, indicating the analytical sensitivity is enhanced. To understand the underlying mechanism of the delaying effect of the sponge shunt, we mathematically simulated the fluid flow in LFA and calculated the delay time as induced by adding sponge shunt. A good agreement between the experiment and simulation data is observed (Fig. 2G), where a thicker sponge shunt will produce longer delay due to the larger flow resistance as descripted in Eq. 5.

**Figure 3:**
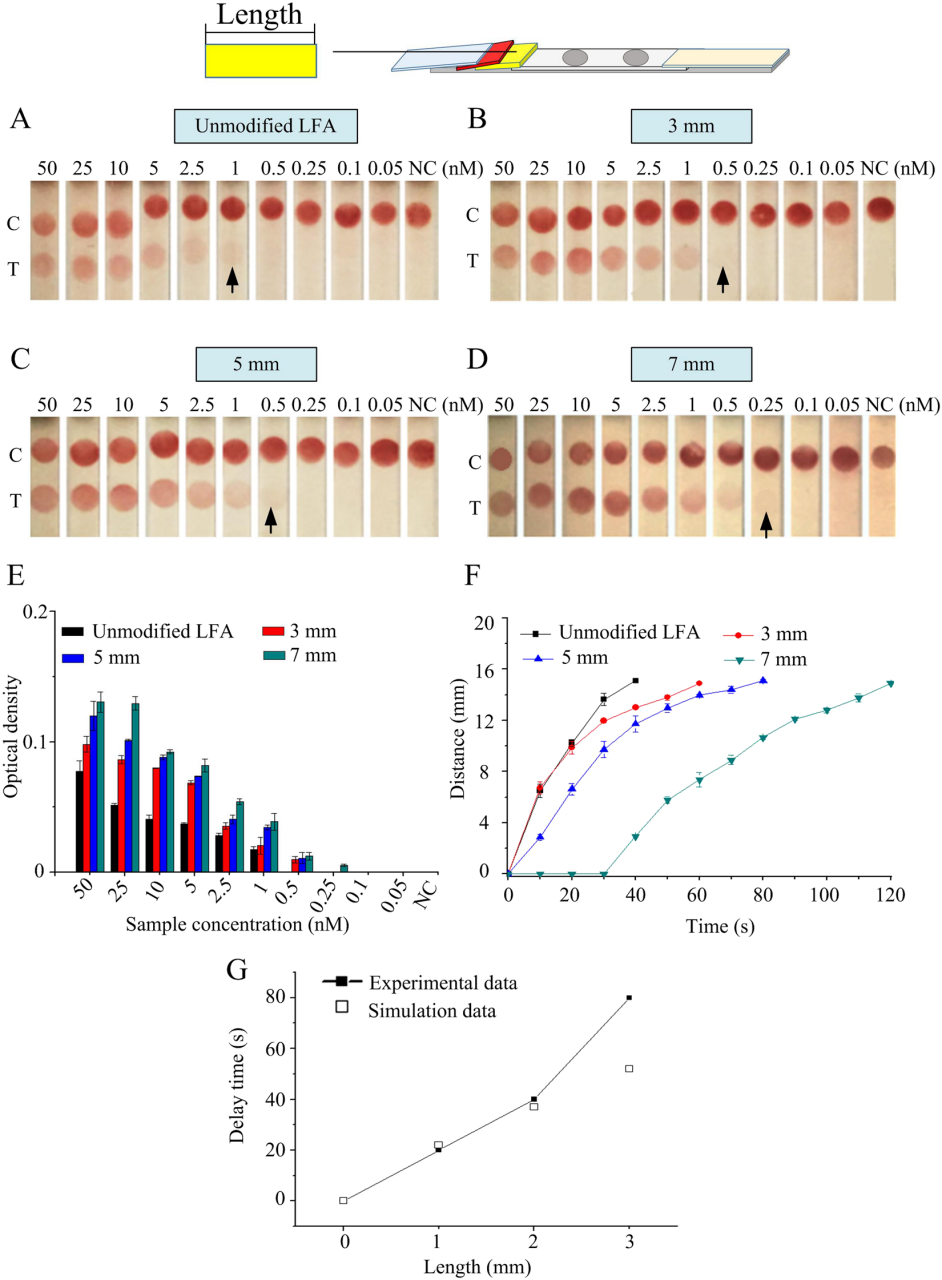
Analytical sensitivity improvement of LFA by different lengths of sponge. The detection limit of unmodified LFA is 1 nM (**A**), 3 mm of sponge length is 0.5 nM (**B**), 5 mm of sponge length is 0.5 nM (**C**) and 7 mm of sponge length is 0.25 nM (**D**). (**E**) The optical density of LFA test line under different lengths of sponge. (**F**) The wicking distance on the NC membrane with time at different sponge shunt lengths. (**G**) The relationship between the experimental data and simulation data. Three replicates were performed for the optimization experiments for high concentrations in the range of 2.5–50 nM (*N* = 3), while eight replicates were performed for lower concentrations in the range of 0.05–1 nM (*N* = 8).

With 3 mm thick and 50°contact angle sponge shunt, different lengths of the shunt (*i*.*e*., 3–9 mm) were also changed to further improve the LFA analytical sensitivity (Fig. 3). The results indicate that the detection limit of the unmodified LFA is 1 nM (Fig. 3A), which is improved to 0.5 nM in LFA with 3 mm and 5 mm long shunt (Fig. 3B,C) and further to 0.25 nM in LFA with 7 mm long shunt (Fig. 3D). The visual observation was also confirmed by the quantification of the signal density in the test line (Fig. 3E). The longer sponge shunt (9 mm) was also tried, the result shows that the liquid cannot flow through NC membrane. The wicking distance on the NC membrane with time was also measured, the result indicates that the wicking distance gradually decreases with the increase of shunt length within the same time (Fig. 3F), because it is mainly attribute to the increased flow resistance (Eq. 6) which is caused by the long sponge. The time for wicking the whole NC membrane with 7 mm length sponge shunt is delayed for 80 seconds over the unmodified LFA, which is also confirmed by our modeling (Fig. 3G).

**Figure 4:**
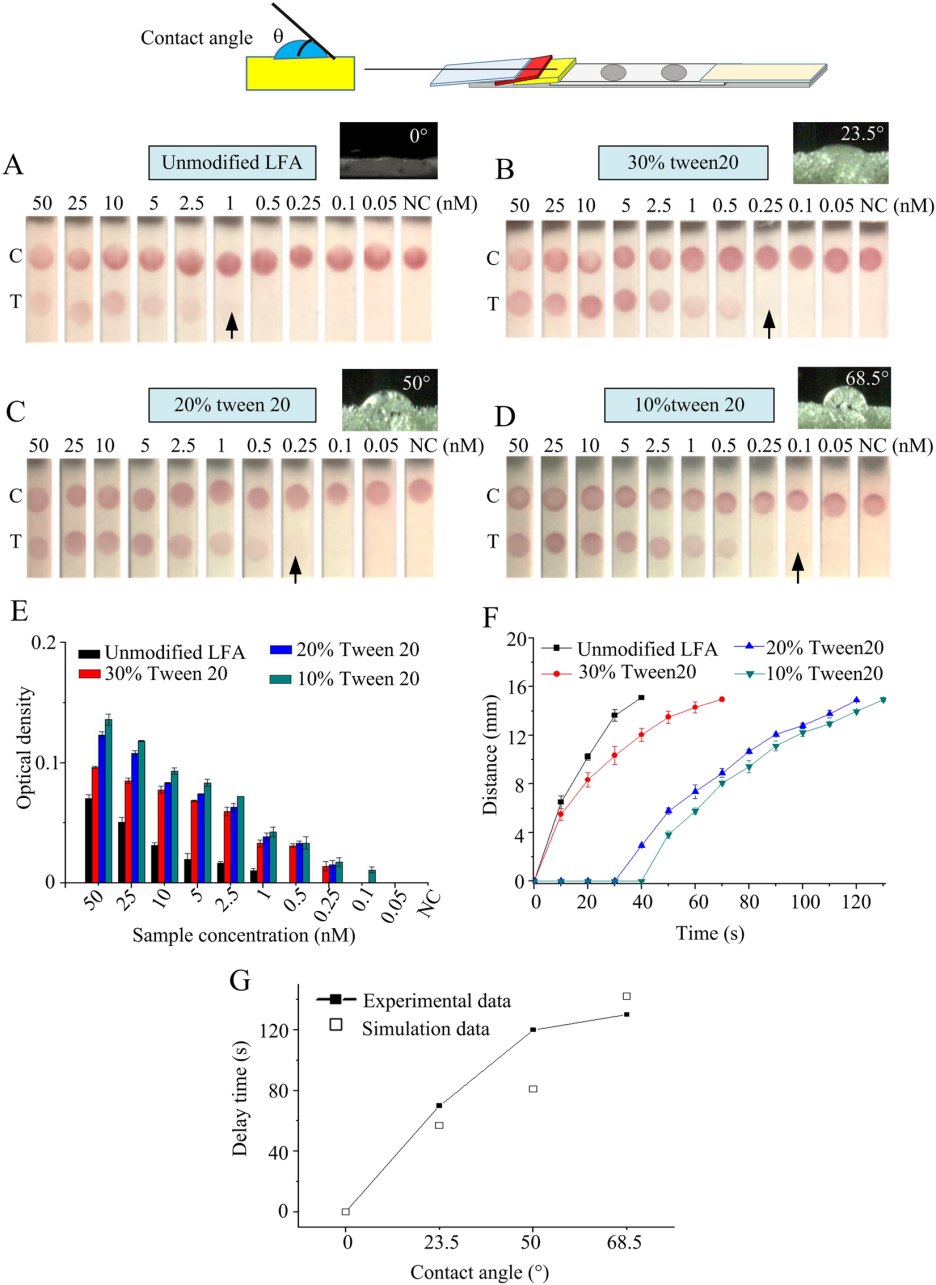
Analytical sensitivity improvement of LFA by different contact angles of liquid on the sponge. The detection limit of unmodified LFA is 1 nM (**A**), 23.5° (30% Tween 20) of contact angle is 0.5 nM (**B**), 50° (20% Tween 20) of contact angle is 0.25 nM (**C**) and 68.5° (10% Tween 20) of contact angle is 0.01 nM (**D**). (**E**) The optical density of LFA test line under different contact angles of liquid on the sponge. (**F**) The wicking distance on the NC membrane with time at different contact angles sponge shunt. (**G**) The relationship between the experimental data and simulation data. Three replicates were performed for the optimization experiments for high concentrations in the range of 2.5–50 nM (*N* = 3), while eight replicates were performed for lower concentrations in the range of 0.05–1 nM (*N* = 8).

Since the hydrophobicity-hydrophilicity of porous material can change the capillary flow of liquid in the porous structure^29^, the hydrophobicity of sponge was further changed by tuning the contact angle of sponge to further increase the LFA analytical sensitivity using sponge shunt with 3 mm thickness and 7 mm length, Fig. 4. To tune the hydrophobicity-hydrophilicity of sponge, sponge was treated using Tween 20 with different concentrations (30%, 20%, 10%, 5%), and the contact angle of sponge was changed from 23.5°, 50° and 68.5° to 102°, respectively. From the results, in comparison with the detection limit of 1 nM in unmodified LFA, the detection limit is improved to 0.25 nM in LFA with sponge with contact angle of 23.5° and 50° (Fig. 4B,C) and further to 0.1 nM in LFA with sponge with contact angle of 68.5° (Fig. 4D), representing an overall 10-fold analytical sensitivity enhancement. Such enhancement was also confirmed by the optical density of LFA test line (Fig. 4E). If the contact angle is further increased (*i*.*e*., 5% Tween 20), the liquid cannot flow through the NC membrane, because the hydrophobic of sponge is increased and capillary force decreased (Eq. 7). Likely, the wicking distance on the NC membrane gradually decreases with the increase of contact angle within the same time (Fig. 4F), that is because the capillary force of the sponge shunt is decreased (Eq. 7). The time for wicking the whole NC membrane with 68.5° contact angle is delayed for 90 seconds over the unmodified LFA, which was confirmed by our modeling (Fig. 4G). Collectively, the results indicate that our assay can achieve 10-fold analytical sensitivity improvement for detection of HBV synthesis target by simply integrating sponge shunt with 3 mm thickness, 7 mm length and contact angle of 68.5° into convectional LFAs, because the fluid flow rate is reduced and the reaction time between targets in sample and AuNP-DPs is increased. In this study, the sponge porosity is 50%, which could improve the analytical sensitivity of LFA. However, the porosity may change for sponge from different commercial manufactures, which may have different effect on the fluid flow rate.

**Figure 5:**
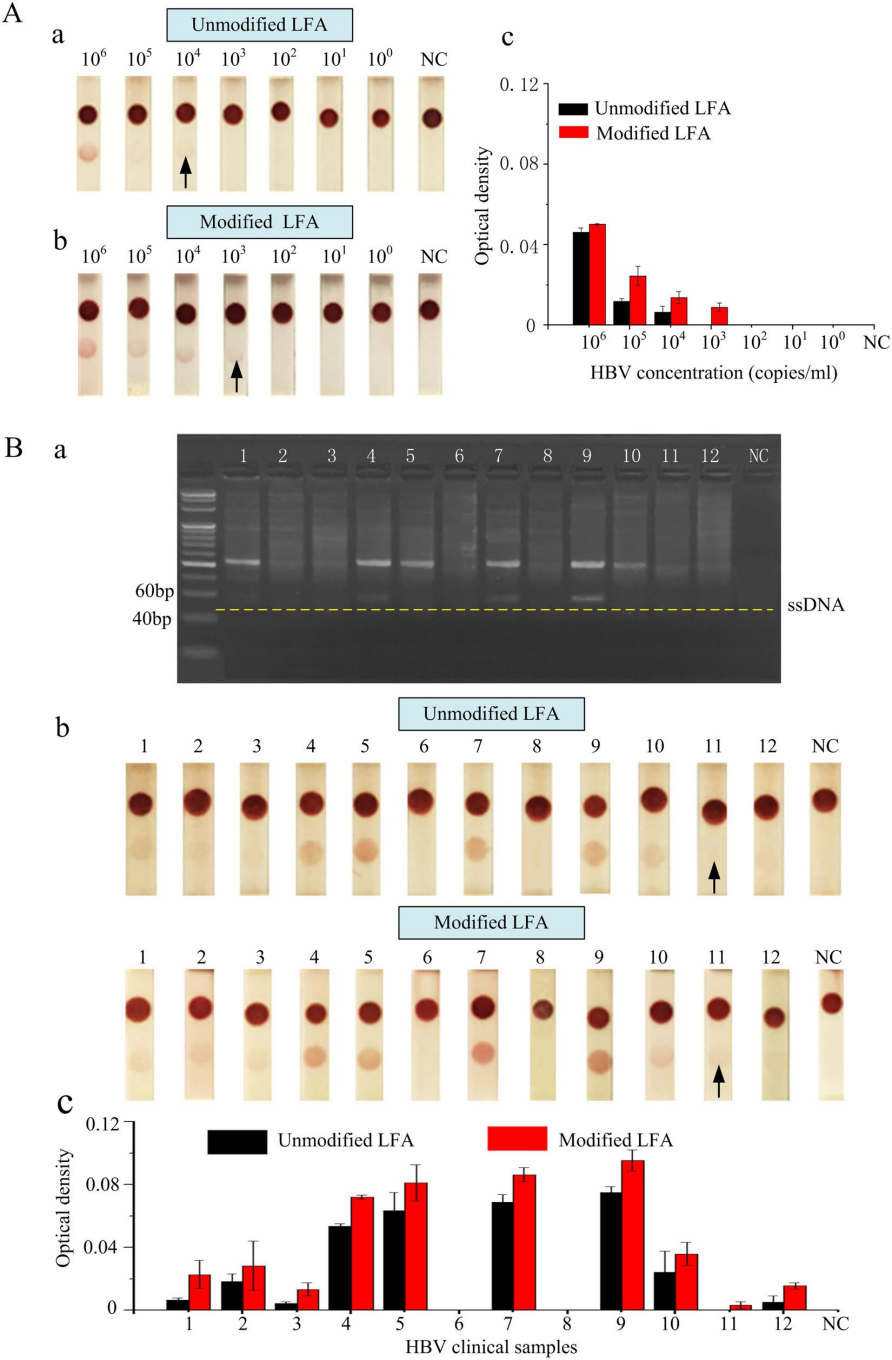
HBV clinical sample detection using unmodified LFA and modified LFA. (**A**) (a) HBV positive serum was detected by normal model, (b) HBV positive serum was detected by modified model, (c) the optical density of LFA test line in normal model and modified model. (**B**) 12 of clinical blood samples were detected by (a) the electrophoresis, (b) the normal model and the modified model, (c) the optical density of LFA test line in unmodified model and modified model. Eight replicates were performed for lower concentrations in the range of 10^2^–10^5^ copies/ml (*N* = 8).

To further verify the analytical and clinical sensitivity enhancement of our proposed LFAs, HBV positive serum and clinical samples were also tested, respectively. For this, FTA card was utilized to extract DNA from serum and clinical blood sample, asymmetric tHDA with 60 min was used to obtain the single-strand nucleic, and nucleic acid-based LFA with 15 min was used to detect the amplification product. The positive serum with HBV concentration from 10^0^ copies/ml to 10^6^ copies/ml was firstly utilized to determine the detection limit using unmodified LFA and our modified LFA (Fig. 5A), respectively. The results show that the detection limit of our modified LFA is 10^3^ copies/ml (Fig. 5A(b)), which is enhanced 10-fold as compared to 10^4^ copies/ml achieved by unmodified LFA (Fig. 5A(a)). Then, 12 HBV clinical blood samples were detected. Before LFA testing, these samples were tested by electrophoresis, these results show clearly visible bands whereas No. 6, No. 8 and negative control show no bands (Fig. 5B(a)). For the unmodified LFA, the results indicate that the color and the optical density of different concentrations of clinical samples are differences in Fig. 5B(b,c). Meanwhile, No. 6, No. 8 and No. 11 samples show negative results, because these HBV concentrations are lower than the detection limit of unmodified LFA (10^4^ copies/ml). For the modified LFA, the color and the optical density of 12 clinical samples are higher than that of unmodified LFA, whereas No. 11 sample shows positive result, indicating that the HBV concentration is 10^3^ copies/ml, and the HBV concentrations of No. 6 and No. 8 samples are lower than 10^3^ copies/ml, which are consistent with the concentration of clinical detection. Analyzing all of the results together according to the reported study^30^, the analytical sensitivity of unmodified LFA and modified LFA for HBV detection is 75% and 100%, respectively. Meanwhile, the clinical sensitivity of electrophoresis, unmodified LFA and modified LFA for HBV detection is 100%, 90% and 100%, respectively. Finally, these results prove that this prototype could detect clinical sample with concentrations of HBV as low as 10^3^ copies/ml.

Compared with the reported flow control studies^18, 21^, our proposed method is low-cost, simple and easy to fabricate, which could be easily integrated into one single paper-based biosensor for different targets testing such as disease diagnosis, food safety detection and environment monitoring, expanding its application in resource-poor settings. We envision that our propose assay has great potential for nucleic acid testing at point of care.

## Conclusion

In this study, we developed a LFA assay to decrease fluid flow rate to improve the analytical sensitivity of LFA by integrating sponge shunt into conventional LFA. With an optimal thickness, length and contact angle of sponge shunt, the fluid flow rate could be decreased and 10-fold LFA analytical sensitivity improvement has been achieved. Additionally, this assay can be combined with FTA card and tHDA technique to successfully detect HBV clinical sample with the detection limit of as low as 10^3^ copies/ml, demonstrating its potential for widespread POCT application such as diseases diagnostics, food safety control and environment monitoring.

## Electronic supplementary material

Improved Analytical Sensitivity of Lateral Flow Assay using Sponge for HBV Nucleic Acid Detection
