# Supplementary material for: Improved Analytical Sensitivity of Lateral Flow Assay using Sponge for HBV Nucleic Acid Detection
Source: Sci Rep. 2017 May 2;7:1360. doi: 10.1038/s41598-017-01558-x (PMC5431006; doi:10.1038/s41598-017-01558-x)
Supplement: Supplementary file 1 — Improved Analytical Sensitivity of Lateral Flow Assay using Sponge for HBV Nucleic Acid Detection [file 41598_2017_1558_MOESM1_ESM.pdf]

Improved Analytical Sensitivity of Lateral Flow Assay using Sponge for HBV Nucleic Acid Detection

Ruihua Tang[a,b,c], Hui Yang[a,b#], Yan Gong[c,d,e], Zhi Liu[c,f], Xiujun Li[g], Ting Wen[e], ZhiGuo Qu[f], Sufeng Zhang[h], Qibing Mei[a,b], Feng Xu[c,d#]

[a] *School of Life Sciences, Northwestern Polytechnical University, Xi'an 710072, P.R. China*

[b] *Key Laboratory for Space Bioscience and Biotechnology, Northwestern Polytechnical University, Xi'an 710072, P.R. China*

[c] *Bioinspired Engineering and Biomechanics Center (BEBC), Xi'an Jiaotong University, Xi'an 710049, P.R. China*

[d] *The Key Laboratory of Biomedical Information Engineering of Ministry of Education, School of Life Science and Technology, Xi'an Jiaotong University, Xi'an 710049, P.R. China*

[e] *Xi'an Diandi Biotech Company, Xi'an 710049, P.R. China*

[f] *Key Laboratory of Thermo-Fluid Science and Engineering of Ministry of Education, School of Energy and Power Engineering, Xi'an Jiaotong University, Xi'an 710049, P.R. China*

[g] *Department of Chemistry, University of Texas at El Paso, 500 West University Ave, El Paso, Texas 79968, USA*

[h] *College of Bioresources Chemical and Materials Engineering, Shaanxi University of Science and Technology, Xian 710021, China*

[#] *Corresponding authors: kittyyh@nwpu.edu.cn, fengxu@mail.xjtu.edu.cn*

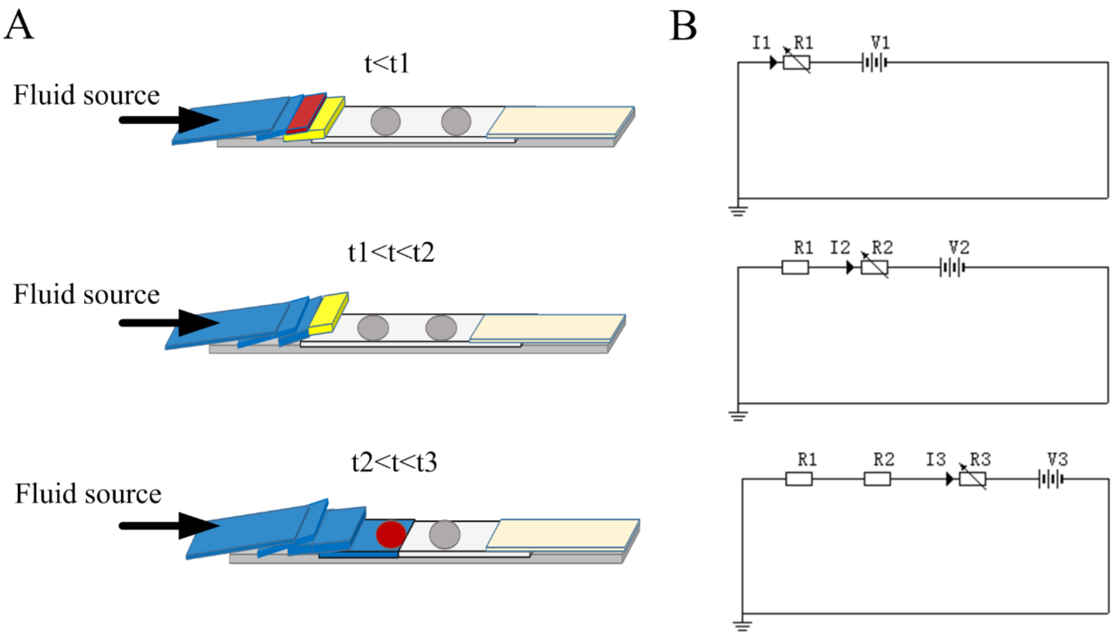

Figure S1. The flow process of the liquid. A: the fluid flow process of the LFA at different the locations of the fluid front. **B**: the electrical circuits of the LFA at different the locations of the fluid front.

Table S1. The primers and LFA probe of HBV preS1 protein.

Primer or Probe Type	Sequence(5'-3')
Forward primer	TACAAAACCTACGGACGGAAACTGC
Reverse primer	AACTGAGCCAAGAGAAACGGACTGA
Control probe	CCTGTATTCCCATCCCAT
Capture probe	ATAGGTATTTTGCGAAAG
Detection probe	ATGGGATGGGAATACAGG

Table S2. Parameters of materials.

**Parameters**	Conjugate pad	Sponge	NC membrane
Length (mm)	10.0	7.0	20.0
Width (mm)	3.0	3.0	3.0
Thickness (mm)	0.2	3.0	0.01
Pore radius (μm)	7.23	20.1	2.73
Porosity	0.38	0.5	0.44

Permeability (m^2)	5.64×10^{-14}	9.18×10^{-14}	1.12×10^{-14}
Contact angle (°)	0	0	0

Table S3. Parameters of liquid

Parameters	Value
Density (kg/m^3)	1000.0
Viscosity (Pa s)	1004.0×10^{-6}
Surface tension (N m)	7.269×10^{-4}

Detection of the concentration of clinical samples

In clinical sample experiment, Blood samples were collected from 12 patients with clinically confirmed HBV infection and quantified using qPCR according to the published protocol [1]. The initial concentration of positive HBV serum was 10^7 copies/ml which was diluted with negative serum to the concentration of 10^6 copies/ml, 10^5 copies/ml, 10^4 copies/ml, 10^3 copies/ml, 10^2 copies/ml, 10^1 copies/ml, 10^0 copies/ml to create a standard curve. 30 µL of serum was utilized for DNA extraction. Then, the template was used for qPCR. CT values were plotted against log (copy number) by ABI 7500 Fast Real-time PCR system software to obtain a standard curve. Slope parameters were estimated by linear regression of CT values vs. log (copy number). For the slope estimates, 95 % Confidence Intervals were calculated using OriginPro 8.0 software. The slope value was applied to calculate the PCR efficiency. Through the results, the amplification curve **(Fig. S2A)** and the standard curve **(Fig. S2B)** were created. According to the standard curve, the concentration of clinical sample was provided in Table S4.

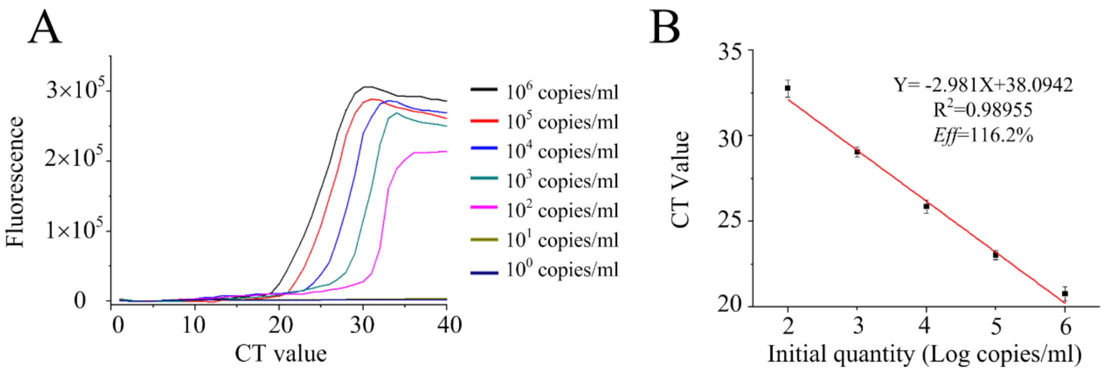

Figure S2. A: Amplification curve of standard positive sample from 10^0 to 10^6 copies/mL; **B:** Standard curve of average CT vs initial quantity (Log copies/ml) obtained with positive control plasmid with 10^0 to 10^6 copies/ml.

TableS4. The concentration of clinical sample.

Number	Concentration (copies/ml)
1	5×10^4
2	7×10^4
3	3×10^4
4	1.2×10^6
5	2.8×10^6
6	Normal
7	1.8×10^7
8	Normal
9	3×10^7
10	3×10^5
11	1×10^3
12	4×10^4

Reference

1 Simon Siu-Man Sum, D. K.-H. W., 1 Man-Fung Yuen,1 He-Jun Yuan,2 Jian Yu,3 & Ching-Lung Lai, D. H., 3 and Linqi Zhang3. Real-Time PCR Assay Using Molecular Beacon for Quantitation of Hepatitis B Virus DNA. *Journal of clinical microbiology* **42**, p. 3438-3440, doi:10.1128/JCM.42.8.3438-3440.2004 (2004).
